# DeepRTCP: Predicting ATP-Binding Cassette Transporters Based on 1-Dimensional Convolutional Network

**DOI:** 10.3389/fcell.2020.614080

**Published:** 2021-02-01

**Authors:** Zhaoxi Zhang, Juan Wang, Jiameng Liu

**Affiliations:** ^1^School of Computer Science, Inner Mongolia University, Hohhot, China; ^2^Stage Key Laboratories of Reproductive Regulation & Breeding of Grassland Livestock, Hohhot, China

**Keywords:** ABC transporters, deep convolutional neural network, tripeptide composition, cross validation, PSSM

## Abstract

ATP-binding cassette (ABC) transporters can promote cells to absorb nutrients and excrete harmful substances. It plays a vital role in the transmembrane transport of macromolecules. Therefore, the identification of ABC transporters is of great significance for the biological research. This paper will introduce a novel method called DeepRTCP. DeepRTCP uses the deep convolutional neural network and a feature combined of reduced amino acid alphabet based tripeptide composition and PSSM to recognize ABC transporters. We constructed a dataset named ABC_2020. It contains the latest ABC transporters downloaded from Uniprot. We performed 10-fold cross-validation on DeepRTCP, and the average accuracy of DeepRTCP was 95.96%. Compared with the start-of-the-art method for predicting ABC transporters, DeepRTCP improved the accuracy by 9.29%. It is anticipated that DeepRTCP can be used as an effective ABC transporter classifier which provides a reliable guidance for the research of ABC transporters.

## 1. Introduction

The ABC transporter is a member of ATP-binding protein superfamily. The core structures of ABC transporters are two nucleotide-binding domains and two transmembrane domains (Abbas et al., 2015). The nucleotide-binding domain is a conserved domain. It can help the transmembrane domain to perform the function. Subdomains of the nucleotide-binding domain have some conserved sequence motifs with specific functions. The most important motifs are Walker-A motifs and LSGGQ motifs. In the process of molecular transports, the two nucleotide-binding domains bind together. There will be two ATP-binding sites and two hydrolysis sites between the Walker-A motifs of one nucleotide-binding domain and the other nucleotide-binding domain (Chen et al., 2003). The ABC transporter performs its transport functions based on the nucleotide-binding domain and the transmembrane domain. The transport functions of ABC transporters are divided into two types: inward transport and outward transport. The inward ABC transporter exists not only in prokaryotes but also in eukaryotes. It can promote the transport of nutrients such as amino acids and carbohydrates from the extracellular environment into the intracellular matrix, thereby promoting cell growth. Outward ABC transporters, like inward ABC transporters, coexist in prokaryotes and eukaryotes. They can expel antibiotics, fatty acids and other substances that are not conducive to cell growth. Outward ABC transporters help cells to keep non-essential foreign substances or secondary metabolites in a low concentration range, thereby reducing the growth pressure of the cells, maintaining the normal growth of the cells, and greatly improving the survival rate of the cells (Gedeon et al., 2006; Davidson et al., 2008; Cui and Davidson, 2011). Based on these physiological characteristics, the identification of ABC transporters is of great significance not only for the development of biomedicine, but also for the crop cultivation and the microbial industry. Effective and accurate ABC identification methods are urgently needed.

Biological experiments are reliable methods for identifying protein functions. But most of these methods require expensive equipments and long experimental cycles. In addition, the experimental method cannot give priority to a function which needs to be identify urgently (Konc et al., 2013). With the development of sequencing technology, a large number of new protein sequences have been discovered. Biological experiments alone are not enough to meet the growing need for protein function identification. Researchers need a fast and accurate method to help identify protein functions. In recent years, predicted methods for protein functions have been widely used. Predicted methods have improved the efficiency of protein identifications, and its accuracy is also high. Protein function predictions usually use machine learning algorithms (Libbrecht. and Noble, 2015) as the classifiers such as support vector machine (SVM) (Suykens and Vandewalle, 1999), random forest (RF) (Vladimir et al., 2003), naive bayes (NB) (Rish, 2001) and artificial neural network (ANN), and obtain good results. The development of deep learning (Lecun et al., 2015) has further improved the performance of predicted methods (Gligorijevic et al., 2018; You et al., 2018). The most commonly used deep learning algorithm is the deep convolutional neural network (DCNN) (Lecun and Bottou, 1998). It has got good results in both the identification of protein functional sites and the protein function prediction (Kulmanov and Robert, 2019; Zhang and Yu, 2019).

A good feature is also crucial for the protein function prediction. In past studies, researchers usually used information extracted from proteins as features, including protein-protein interactions (Haretsugu et al., 2010; Jiang, 2012), structural information (Zhang et al., 2016; Le et al., 2019), physicochemical property (Cai et al., 2003), amino acid composition (Luo et al., 2010), evolutional information (Mundra et al., 2007), and the combinations of different information mentioned above (Chen et al., 2012, 2013; Song et al., 2014; Zou et al., 2016). Among these features, amino acid composition and evolutional information have been widely used. The amino acid composition is divided into peptide composition, dipeptide composition, and tripeptide composition, etc. The tripeptide composition contains more information than the peptide composition and the dipeptide composition. But the dimension of tripeptide composition is large. The tripeptide composition of amino acids sequence is an 8000-dimensional vector. The tripeptide composition of most proteins is a sparse vector, which affects the use of tripeptide composition in protein function predictions. Lin et al. (2017) divided the 20 amino acids into several pseudo-amino-acids called reduced amino acid alphabet (RAAA). By using RAAA to represent protein sequences, the dimension of the tripeptide composition can be reduced, thereby improving the accuracy of protein function predictions. The position specific score matrix (PSSM) (Michael et al., 1987) contains evolutional information of a protein. It is obtained from the multiple sequence alignment (MSA). PSSM contains statistical information about the distribution of residues at different positions in a MSA. The value in PSSM represents the score that a residue at one position will mutate to another residue during evolution. PSSM contains information about the homologous sequence of the query protein, which is not available in other sequence-based protein features. It has achieved good results in protein function predictions (Wang et al., 2018, 2019; Gao et al., 2019).

**Figure 1:**
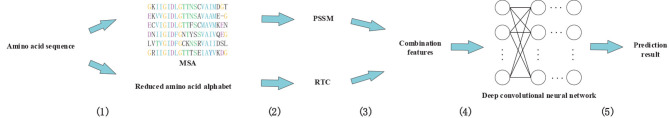
An example using DeepRTCP to predict ABC transporters. (1) Multiple sequence alignments were built for query proteins, and amino acid sequences were replaced with reduced amino acids sequences. (2) The PSSM of the query protein was calculated from multiple sequence alignment, and the RTC was extracted from the reduced amino acids sequence. (3) PSSM and RTC combine into RTCP. (4) Deep convolutional neural network uses RTCP as input to train the classifier. (5) The output of the convolutional neural network passes through a binary classifier to get the prediction result.

In this study, we proposed a novel method called DeepRTCP. It applies the 1-dimensional DCNN and a feature combined of the PSSM and RAAA based tripeptide composition to predict ABC transporters. This experiment used a dataset named ABC_2020. Through experimental comparison, we chose the feature based on surface tension and solvent solubility, and chose a 7-layers DCNN as the classifier. Finally, we compared DeepRTCP with the state-of-the-art method for predicting ABC transporters. The results show that DeepRTCP is better than the existing method in all evaluation indicators used in this article. The overall process of using DeepRTCP to predict ABC transporters is shown in Figure 1.

## 2. Materials and Methods

### 2.1. Dataset

This experiment used a dataset named ABC_2020. It includes 2,105 positives and 2,105 negatives. The positives were download from the Swiss-prot database of Uniprot (Amos et al., 2009) by using a key word "ABC transporter." We used CD-HIT to remove redundant sequences in the downloaded data. We hope to keep a large number of samples while reducing the impact of redundant sequences on the model, so that our model can be fully trained. So we chose 0.6 as the E-value of CD-HIT. We selected the protein families in Pfam (Finn et al., 2014), which do not contain the proteins in positives. Then we took the longest protein sequence in each protein family as a negative. We got 9,736 negatives and randomly selected 2,105 sequences from these negatives as the negative set of ABC_2020.

### 2.2. Feature Extraction

We proposed a novel RAAA based on the physicochemical property of amino acids. The research used four physicochemical properties, including hydrophobicity (HP), surface tension (ST), solvent solubility (SS), and charged polarity (CP). Each physicochemical property divides amino acids into three classes (as shown in Table 1). We regarded the intersection set of a class of amino acid based on a type of physicochemical property and a class of amino acid based on another type of physicochemical property as a reduced amino acid. Therefore, every two types of physicochemical properties can determine an expression of RAAA. Table 2 shows six RAAA representations: HP_ST, HP_SS, HP_CP, ST_SS, ST_CP, and SS_CP. We replaced the amino acid sequence with a RAAA sequence. The RAAAs of HP_ST, HP_SS, HP_CP, ST_SS, ST_CP, and SS_CP are composed of 7, 5, 5, 7, 8, and 6 reduced amino acids, respectively. We counted the frequency of different tripeptides in the RAAA sequence to obtain the RAAA based tripeptide composition (RTC). The RTC used in this experiment is a 1-dimensional vector. Comparing with amino acid based tripeptide composition (TC), RTC further adds the information of the physicochemical property of the amino acid.

**Table 1:** Classification of amino acids based on different types of physicochemical properties.

**Physicochemical properties**	**Class 1**	**Class 2**	**Class 3**
Hydrophobicity	RKEDQN	GASTPHY	CVLIMFW
Surface tension	GQDNAHR	KTSEC	ILMFPWYV
Solvent solubility	ALFCGIVW	KTSEC	MPSTHY
Charged polarity	LIFWCMVY	PATGS	HQRKEND

**Table 2:** RAAAs based on different types of physicochemical properties.

**Combination of different classes**	**HP_ST**	**HP_SS**	**HP_CP**	**ST_SS**	**ST_CP**	**SS_CP**
Class-1-1	RQND				GA	ICWFLV
Class-1-2	EK	NEQRDK		AG	NQDR	AG
Class-1-3			RKDQEN	RNDQH	H	
Class-2-1	AGH	AG	Y	C	C	
Class-2-2	ST		GPSTA	ST	EK	
Class-2-3	PY	SPYHT	H	KE	TS	RDENQK
Class-3-1		CFIVWL	LFVCIWM	MYVLFWI	FLWIV	MY
Class-3-2	C			P		SPT
Class-3-3	WIFLMV	M			MYP	LH

*Class-i-j represents a class of reduced amino acid that determined by the intersection set between Class-i of one physicochemical property and Class-j of another physicochemical property*.

For a protein *P*, the PSSM of *P* was obtained by PSI-BLAST (Altschul et al., 1997). The database used in PSI-BLAST is Swissprot which can be download from ftp://ftp.ncbi.nih.gov/blast/db/FASTA/. The PSSM contains the evolutional information of a protein. It is a L*20 matrix, where *L* is the length of *P*. The matrix is shown as follows:

(1) $$\begin{array}{l} PSSM={\left[\begin{array}{ccc} a_{1,1} & \ldots & a_{1,L}\\ \vdots & \ddots & \vdots \\ a_{20,1} & \ldots & a_{20,L} \end{array}\right]}_{20\times L} \end{array}$$

where *a*_*i, j*_ represents the score that the *i*-th residue in *P* evolves into an amino acid *j*. We used the following formulas to convert PSSM into a 20-dimensional vector:

(2) $$A_{i,j}=\frac{1}{1+e^{-a_{i,j}}}$$

(3) $$PSSM-feature=\left\{\frac{\sum_{i=1}^{L}A_{i,j}}{L}|j=1,2\ldots 20\right\}$$

We spliced the feature vectors of RTC and PSSM together to form a new feature called RTCP, and used RTCP as the input of the classifier.

### 2.3. Classifier

**Figure 2:**
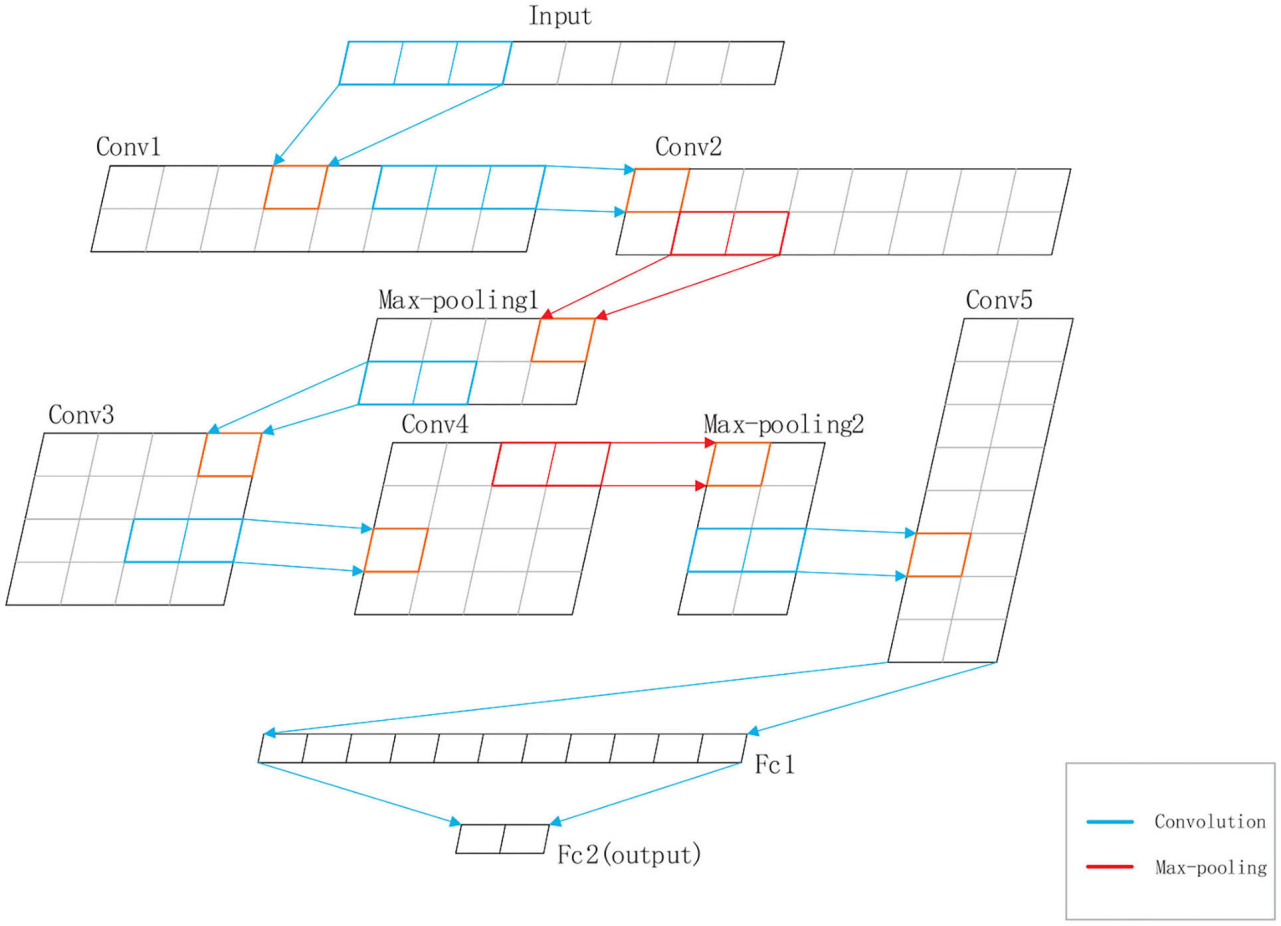
The structure of the DCNN used in DeepRTCP. The DCNN has five convolutional layers: Conv1, Conv2, Conv3, Conv4, and Conv5, five batchnormalization layers, two max-pooling layers: Max-pooling1 and Max-pooling2, and two fully connected layers: Fc1 and Fc2. Fc2 is the output layer. The blue line represents the convolution. The red line represents the max-pooling.

DeepRTCP uses SVM and DCNN as classifiers. SVM is used for choosing the optimal RTCP. DCNN uses the optimal RTCP as input to determine whether a protein is an ABC transporter. SVM is a powerful and effective machine learning algorithm. We used the RBF kernel SVM in this study. The penalty parameter and the gamma were set into 10^5^ and “auto,” respectively. DCNN is a heuristic algorithm that imitates the local receptive field of biological neurons. We used a 1-dimensional DCNN in this work. Figure 2 shows the architecture of the DCNN, which consists of 5 convolutional layers, 5 batchnormalization layers, 2 max-pooling layers and 2 fully connected layers. The convolutional layer extracts important local information from the input features. The fully connected layer is equivalent to a classifier. It uses the information extracted by the convolutional layer to classify the input protein. If we have an input *x* of length *L* and a kernel function *f*(*x*), the output of the convolution operation is defined as:

(4) $$\begin{array}{l} y=\left[a_{l},a_{2},a_{3}\ldots \ldots ,a_{k}\right],k=\frac{L-F}{S} \end{array}$$

(5) $$\begin{array}{l} a_{i}=\overset{F}{\underset{j=1}{\sum }}f\left(j\right)\times x\left(i+j-1\right) \end{array}$$

where *F* is the length of the filter, and *S* is the stride. The *i*-th value of the output vector is obtained by the convolution summation of the *x*[*i*:*i*+*F*−1] and the convolution kernel *f*(*x*).

In order to accelerate the convergence rate of the model, we inserted a batchnormalization layer after each convolutional layer. The batchnormalization layer ensures that the distribution of features in each batch will not change much. We also added max-pooling layers to the network to reduce the redundant information contained in the output of the convolutional layer.

### 2.4. Training

DeepRTCP takes the RTCP as input. The RTCP is a 532-dimensional vector. We used PCA (Belhumeur et al., 1997) to reduce the dimension of RTCP to 80 to optimize the performance of DeepRTCP. In order to fit the model better, we used a learning rate decay strategy. We set the initial learning rate to 10^−3^, and then reduced the learning rate to one-tenth of the original value every 100 epochs. We had trained the model for 1,000 epochs, and the learning rate will continue to decrease until the end of training. DeepRTCP uses Relu as the activation function of hidden layers, Sigmoid as the activation function of the output layer, Adam as the optimizer and binary cross-entropy as the loss function. The strides of convolution and pooling are 2. This experiment used Tensorflow (Rampasek and Goldenberg, 2016) to build the deep learning model and Tensorboard to record the loss and the accuracy of the model. DeepRTCP ran on Nvidia RTX 2070(8G) GPU card. Training time of DeepRTCP was greatly reduced by using the CUDA (Nickolls et al., 2008) framework and the GPU card.

### 2.5. Evaluation Methods

This experiment uses some evaluation indicators that are widely used in protein function predictions, including accuracy (Acc), specificity (Spec), sensitivity (Sens), F-score, and Matthews' correlation coefficient (Mcc) (Matthews, 1975; Shan et al., 2019; Zhang et al., 2019). The formulas of these indicators are as follows:

(6) $$\begin{array}{l} Acc=\frac{TP+TN}{TP+FP+TN+FN} \end{array}$$

(7) $$\begin{array}{l} Spec=\frac{TN}{TN+FP} \end{array}$$

(8) $$\begin{array}{l} Sens=\frac{TP}{TP+FN} \end{array}$$

(9) $$\begin{array}{l} Pre=\frac{TP}{TP+FP} \end{array}$$

(10) $$\begin{array}{l} F-score=\left(1+\beta^{2}\right)\frac{Pre\times Sens}{\beta^{2}\times \left(Pre+Sens\right)} \end{array}$$

(11) $$\begin{array}{l} Mcc=\frac{TP\times TN-FP\times FN}{\sqrt{\left(TP+FP\right)\times \left(TP+FN\right)\times \left(TN+FP\right)\times \left(TN+FN\right)}} \end{array}$$

where TP, FP, TN, and FN represent the rates of true positives, false positives, true negatives, and false negatives, respectively. In the formula of F-score, β measures the importance between Pre and Sens. We set β to 1 which means that Pre is as important as Sens.

We also used ROC curve (Hanley and Mcneil, 1982) and the area under the curve (AUC) to evaluate the performance of different classifiers. If the ROC curve of one method is covered by the ROC curve of another method, the latter is better. But the ROC curves of different methods are usually intersecting. So it is difficult to judge which method is better. So we need to compare AUCs of these methods. The larger the AUC, the better the method.

## 3. Results and Discussion

Firstly, we compared the performances of DeepRTCP when using different RTCPs. Secondly, we compared the RTCP based method with the methods based on TC and PSSM (TCP). Thirdly, we compared the performance among DCNNs with different structures and selected the optimal DCNN for predicting ABC transporters. Fourthly, we compared the performance among different classifiers. Fifthly, we analyzed the predicted false negatives and false positives. Finally, we compared DeepRTCP with the existing method.

### 3.1. Comparison Among Different Types of RTCPs

In order to select the most suitable features, we used SVM to test the performance among six types of RTCPs. The reason for choosing SVM is that it can get results fast. We used PCA to reduce the dimension of the RTCP. We tested the performance among RTCPs with different dimensions. The results were presented in Supplementary Tables 1–6. We compared the performance among different RTCPs. Table 3 shows the comparison results. The ST_SS based RTCP achieved the best performance which mainly related to the nature of the ABC transporters. ABC transporters perform functions on both sides of the cell membrane, which may cause the difference in surface tension and solvent solubility with other proteins.

**Table 3:** Comparison among methods based on different types of RTCP.

**Type of RTCP**	**Acc**	**Spec**	**Sens**	**F-score**	**Mcc**
HP_ST	93.54%	92.21%	94.76%	0.9343	0.8716
HP_SS	93.44%	92.26%	94.28%	0.9334	0.8668
HP_CP	93.23%	93.23%	93.33%	0.9421	0.8619
ST_SS	93.94%	92.45%	94.76%	0.9383	0.8810
ST_CP	93.68%	92.30%	94.28%	0.9357	0.8715
SS_CP	93.32%	92.26%	94.28%	0.9323	0.8691

### 3.2. Comparison With the TCP Based Method

We used PCA to reduce the dimension of the TCP, and then compared its performance with the RTCP's. Table 4 shows that even if PCA is used for dimension reduction, the performance of the TCP based method is still not as good as that of the RTCP based method. Comparing with TC, RTC contains information of the physicochemical property, which makes RTC more efficient than TC for predicting protein functions. The TCP based method achieved the best performance after using PCA to reduce the dimension to 500. This shows that by combining the physicochemical property, RTCP is not only better than TCP in performance, but also has lower dimensions than TCP. This makes the RTCP based method faster and less expensive than TCP based methods.

**Table 4:** Comparison between RTCP based method and TCP based methods.

**Feature**	**Acc**	**Spec**	**Sens**	**F-score**	**Mcc**
TCP_8020	93.25%	91.92%	94.00%	0.9314	0.8507
TCP_1000	93.39%	92.11%	94.59%	0.9330	0.8645
TCP_500	93.49%	92.21%	95.50%	0.9339	0.8738
TCP_200	93.34%	92.01%	94.59%	0.9325	0.8645
TCP_80	93.16%	92.40%	93.69%	0.9309	0.8552
RTCP	93.94%	92.45%	94.76%	0.9383	0.8810

*The number behind TCP represents the dimension of TCP*.

### 3.3. Comparison Among DCNNs With Different Structures

**Figure 3:**
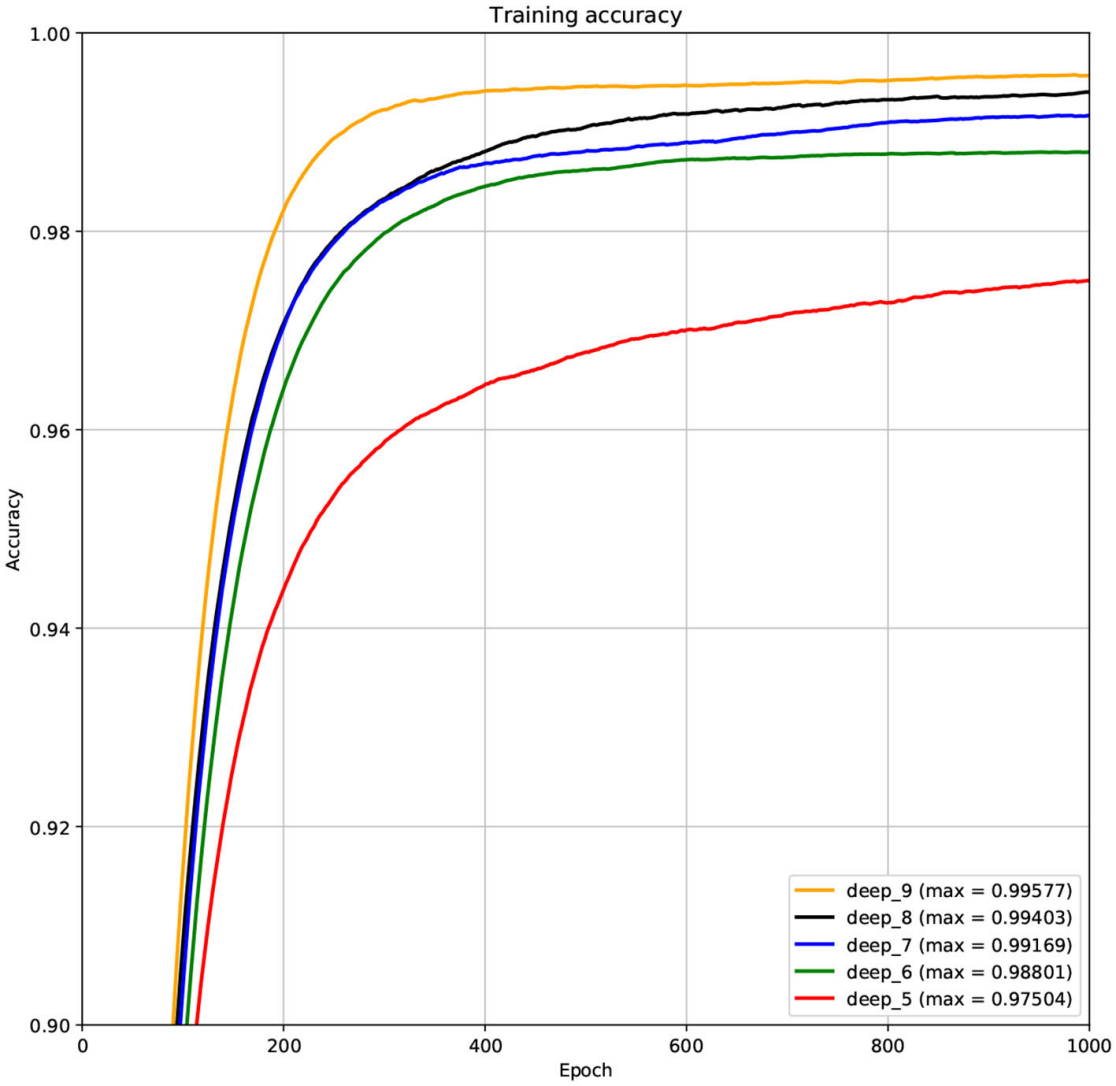
The variation curve of the training accuracy.

**Figure 4:**
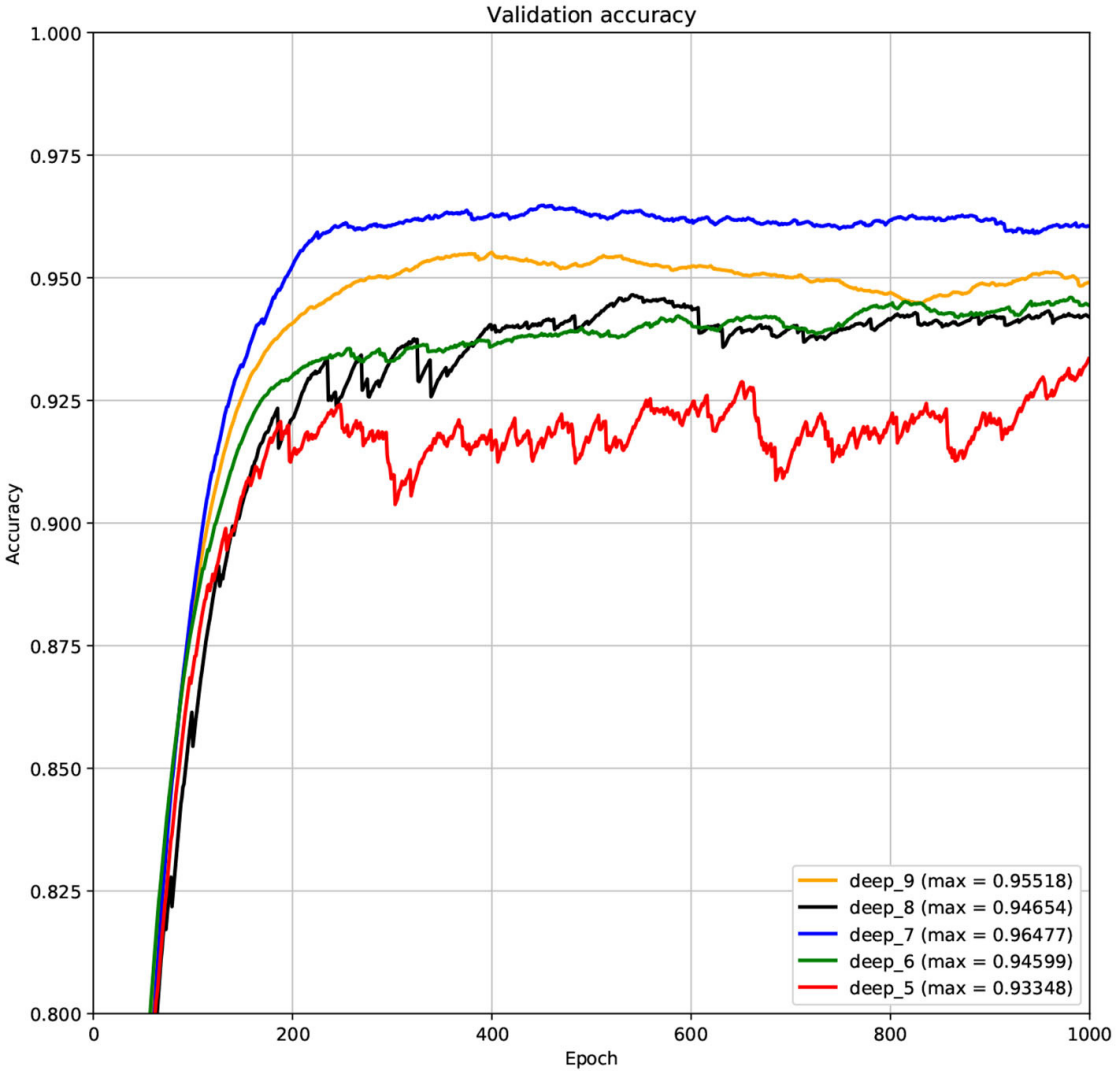
The variation curve of the validation accuracy.

**Figure 5:**
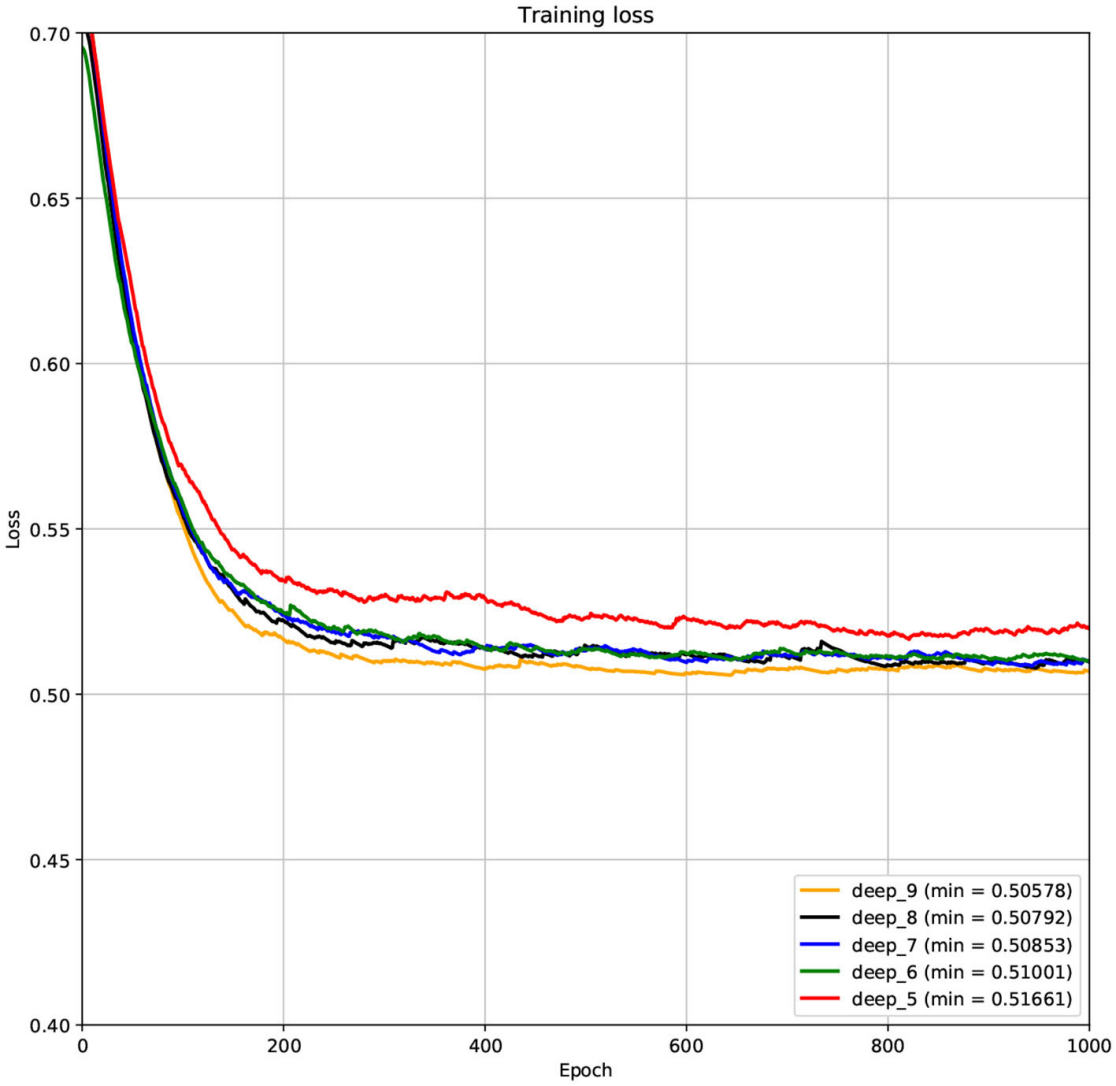
The variation curve of the training loss.

**Figure 6:**
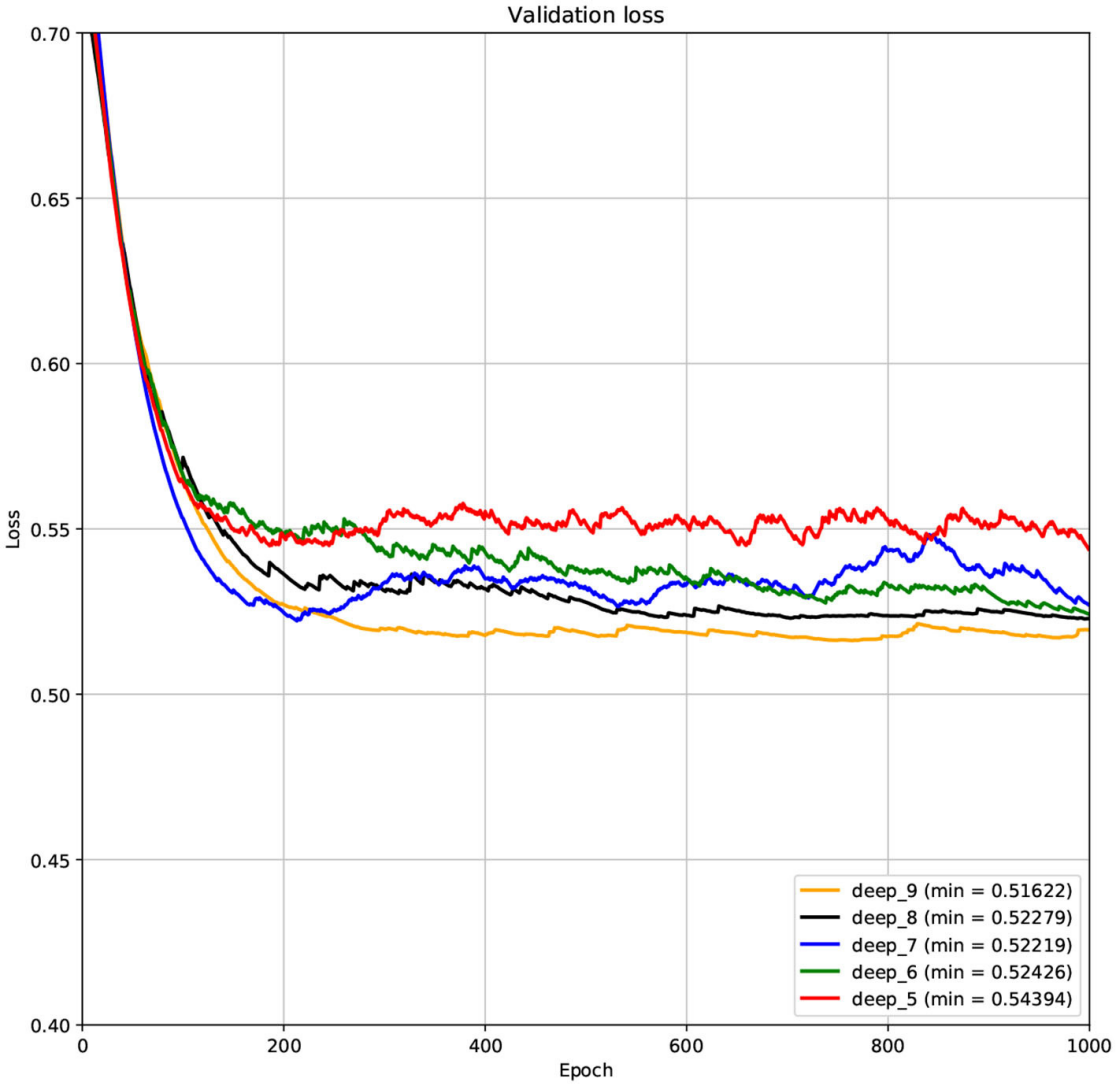
The variation curve of the validation loss.

The structure of DCNN has a great influence on its performance. We compared DCNNs with different depths. The layer numbers of these DCNNs are 5, 6, 7, 8, and 9, respectively. The filter number of these DCNNs is 64. From Figures 3, 4, we can see that the 7-layers DCNN achieve the highest accuracy on the validation set. Comparing with other DCNNs, the 7-layers DCNN has the smallest difference between the training accuracy and the validation accuracy. Therefore, the 7-layers DCNN is more suitable for predicting ABC transporters than DCNNs with other depth. From Figures 5, 6, we can see that using RTCP as the feature, these DCNNs fit around the 400th epoch. The training loss and the validation loss of each DCNN are similar, which indicates the robustness of RTCP. We also compared the performance of 7-layers DCNNs with different filter numbers of 8, 16, 24, 32, 40, 48, 56, 64, 72, and 80, respectively. The result was presented in Supplementary Table 7. After the filters number is greater than 32, the accuracy of the model no longer changes significantly. The 7-layers-32-filters DCNN and the 7-layers-64-filters DCNN had achieved the best validation accuracy. Comparing with the 7-layers-64-filters DCNN, the 7-layers-32-filters DCNN is simpler and less computationally expensive. So the 7-layers-32-filters DCNN was selected as the classifier for this experiment.

### 3.4. Comparison Among Methods Based on Different Classifiers

**Figure 7:**
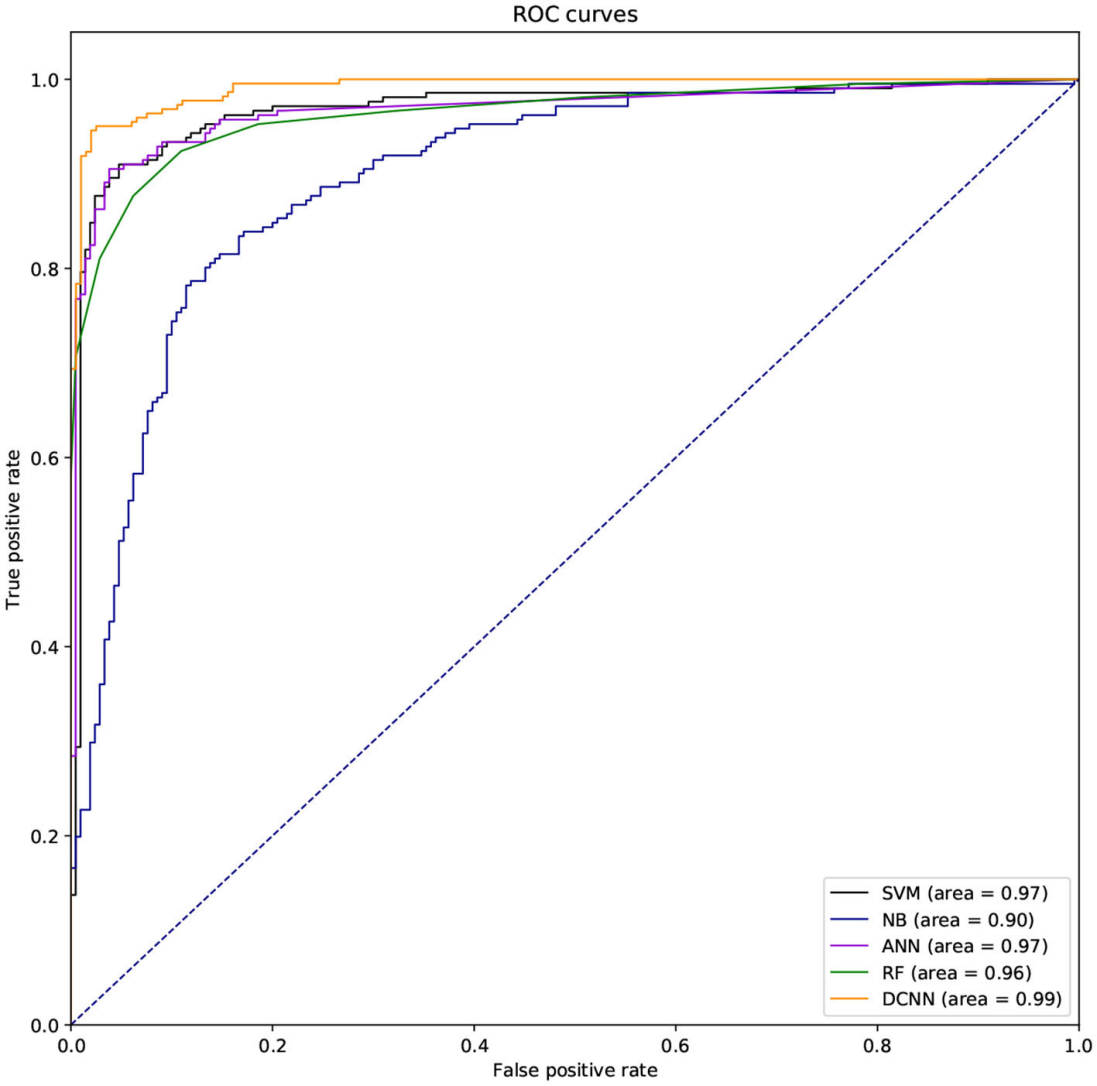
The ROC curves of different classifiers.

We used different classifiers to predict ABC transporters, including SVM, NB, ANN, RF, and DCNN. We used 10-fold cross-validation to evaluate the performance of these classifiers. Table 5 shows the results of the test. Except for NB, the validation accuracies of other classifiers exceeds 90%, and the validation accuracy of DCNN is as high as 95.96%. ANN has the minimum difference of 0.54% between Spec and Sens. DCNN achieved the best F-score and Mcc of 0.9593 and 0.9125, respectively. Then we analyzed the ROC curves of these methods (as shown in Figure 7). It is worth noting that the AUC of DCNN is as high as 0.99. The true positive rate of DCNN is much higher than that of other classifiers when the false positive rate is 0. These results show that DCNN is more suitable for the identification of ABC transporters than other classifiers.

**Table 5:** Performance comparison among different classifiers.

**Classifier**	**Acc**	**Spec**	**Sens**	**F-score**	**Mcc**
SVM	93.94%	92.45%	94.76%	0.9383	0.8810
NB	83.13%	79.52%	86.73%	0.8375	0.6643
ANN	91.69%	91.43%	91.94%	0.9172	0.8337
RF	90.97%	95.71%	86.26%	0.9055	0.8025
DCNN	95.96%	97.14%	94.81%	0.9593	0.9195

### 3.5. Analysis of TP and FP

**Figure 8:**
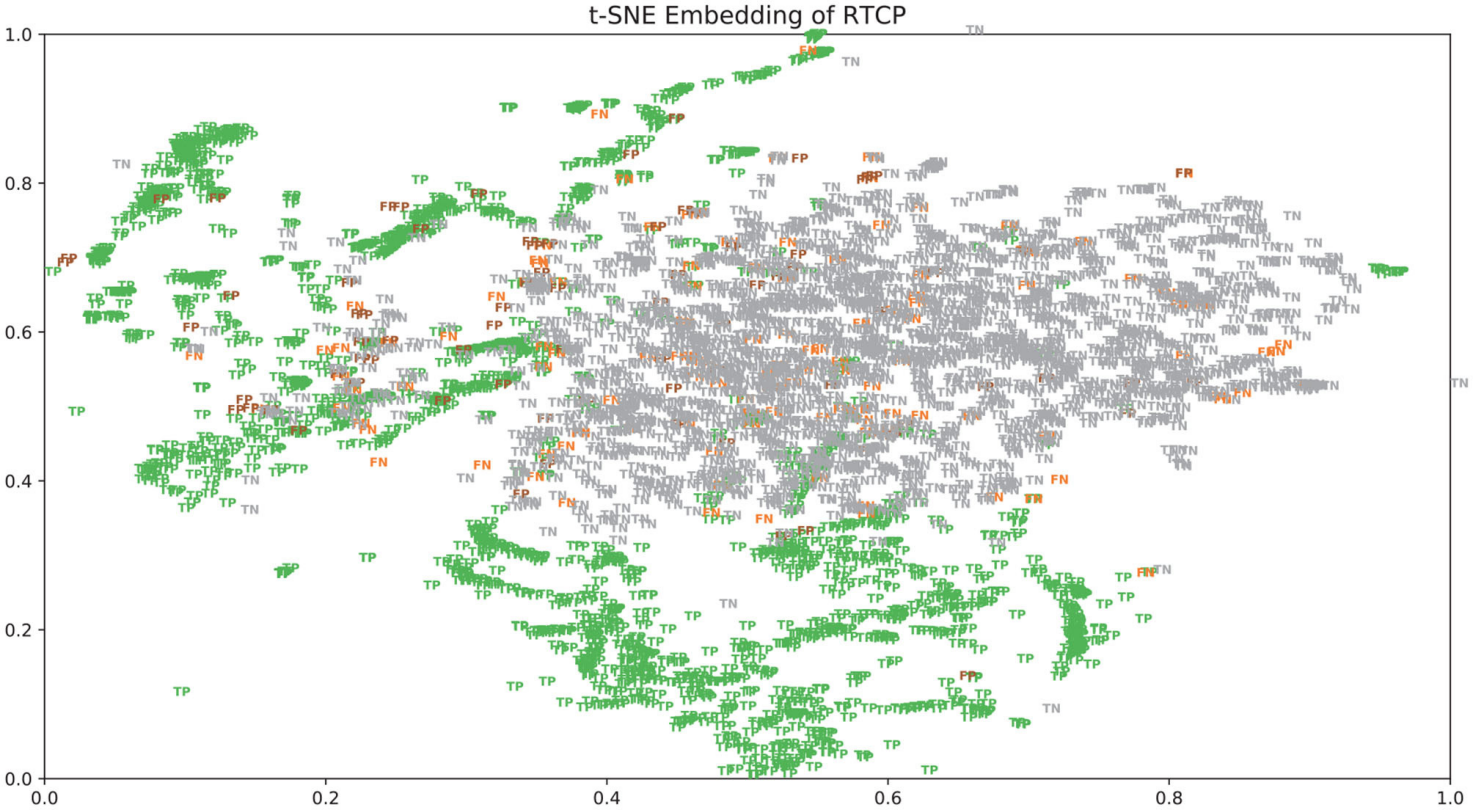
The distribution of the samples in ABC_2020 in two-dimensional space.

**Figure 9:**
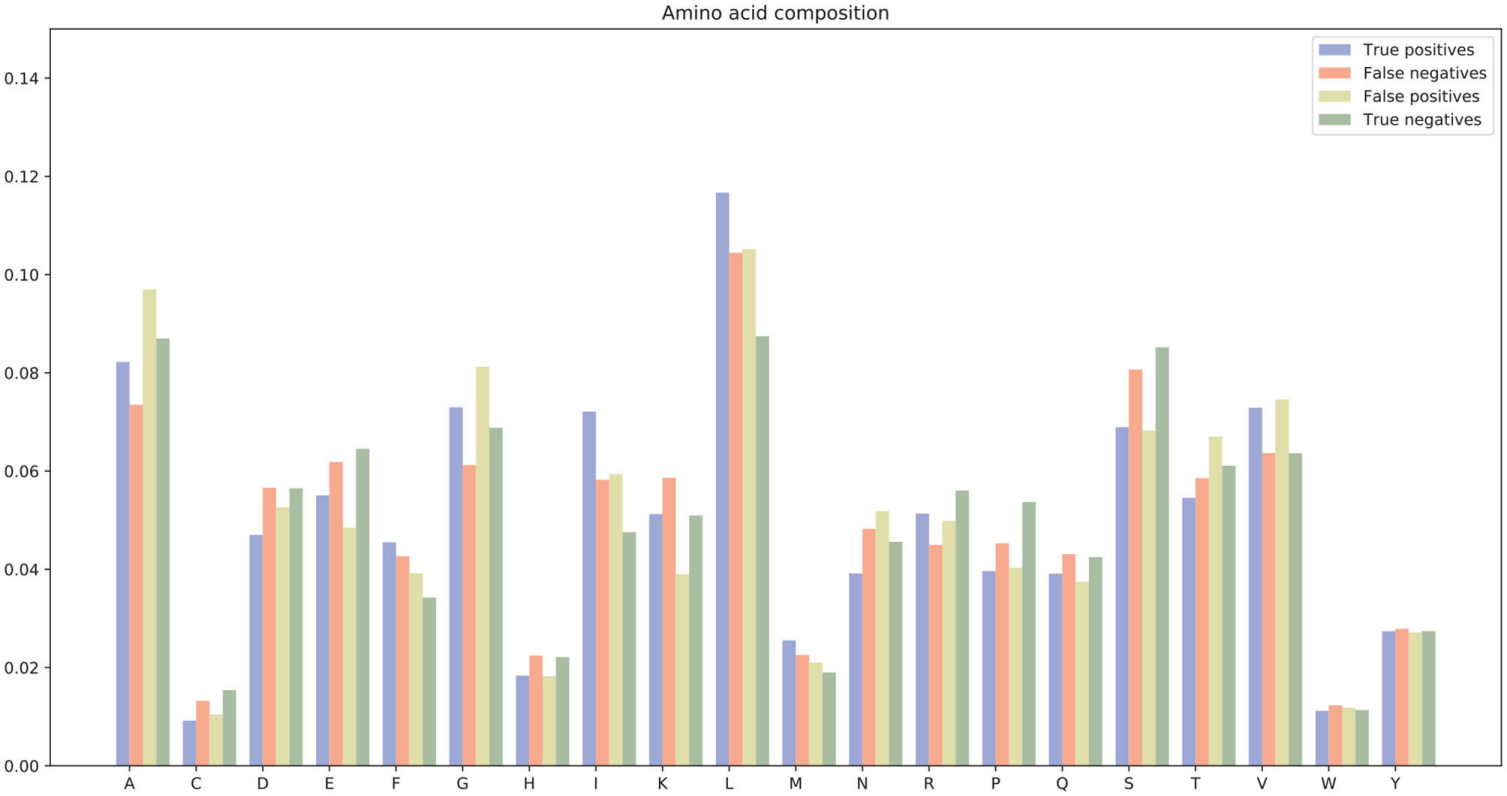
Amino acid composition of samples in ABC_2020.

There were many false positives and false negatives in the prediction results of DeepRTCP. We tried to analyze the reasons why these data were predicted incorrectly. We trained 100 models for each cross-validation, and selected the proteins that were predicted incorrectly more than 50 times as false negatives and false positives. The false negatives and false positives can be obtained from https://github.com/zhichunlizzx/DeepRTCP. We used t-Distributed Stochastic Neighbor Embedding (T-SNE) (Shao et al., 2018) to map the features of the samples in the dataset to two-dimensional space. We founded that in the two-dimensional space, false positives (false negatives) were distributed in the area where the positives were clustered (as shown in Figure 8). We counted the amino acid composition of the samples in ABC_2020 (as shown in Figure 9). We found that the content of histidine, glutamine, and valine in positives (negatives) and false positives (false negatives) are similar, but there are significant differences in the content of histidine, glutamine, and valine in positives (negatives) and false negatives (false positives). This may result in a positive (negative) being incorrectly predicted as a false negative (false positive).

### 3.6. Comparison With Other Method

**Figure 10:**
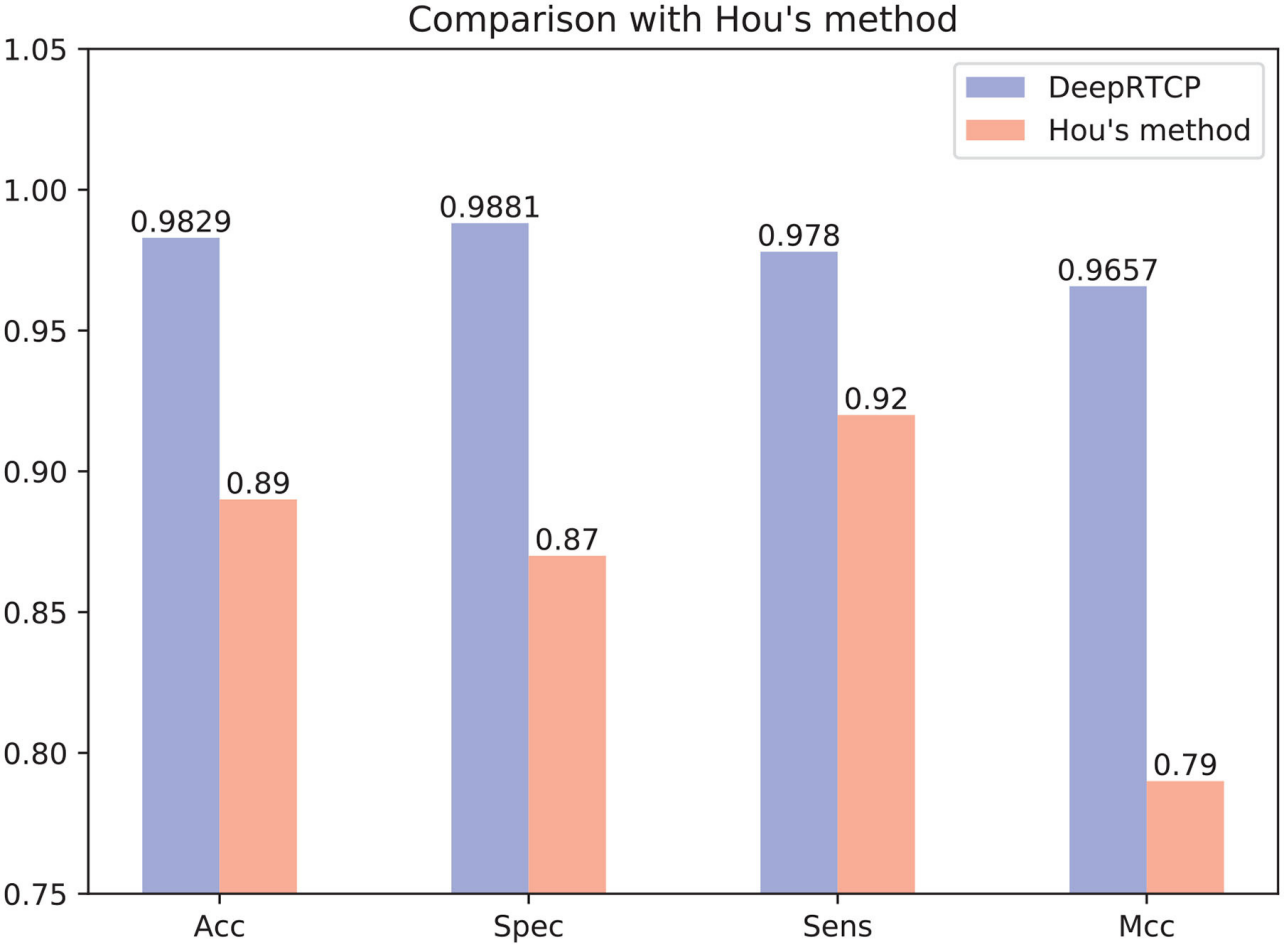
Comparison between DeepRTCP and other method.

In the past study, Hou et al. (2020) used RF and a feature of 188 dimension to predict ABC transporters. We downloaded the dataset provided by Hou, which included 875 positives and 875 negatives. We performed 10-fold cross-validation on DeepRTCP on this dataset and compared the results with Hou's. Since the number of samples in Hou's dataset is smaller than that of ABC_2020, we use a simple DCNN on this dataset. The DCNN includes 4 convolutional layers and 2 fully connected layers. The filter numbers in the four convolutional layers are 16, 16, 32, and 32, respectively. The two fully connected layers contain 13 and 2 neurons, respectively. Figure 10 shows that the average validation accuracy of DeepRTCP is as high as 98.29%. Compared with Hou's method, DeepRTCP improved the Acc by 9.29%, Spec by 12.81%, Sens by 5.8%, and Mcc by 0.1757. In addition, the difference between the Spec and Sens of Hou's method is as high as 5%, which makes Hou's method not well applied in practices. The difference between Spec and Sens of DeepRTCP is about 1%, which is a great improvement to Hou's method. This is mainly due to the effective classifier and feature.

## 4. Conclusion

In this study, we propose a novel method for ABC transporter prediction called DeepRTCP. It uses the DCNN as the classifier. The classifier uses a feature named RTCP which composed of TCP and PSSM. We tested the performance of six types of RTCPs. The results show that the ST_SS based RTCP has the best performance. In the comparison of different classifiers, DCNN achieved the best results that Acc, Spec, Sens, F-score and Mcc were 95.96%, 97.14%, 94.81%, 0.9593 and 0.9195, respectively. Compared with the state-of-the-art method, DeepRTCP improved Acc by 9.29%,Spec by 11.81%, Sens by 5.8%, and Mcc by 0.1757. DeepRTCP can label the ABC transporters faster than traditional biological experiments, and the accuracy of DeepRTCP is also high. DeepRTCP provides a reliable guide for the further research of ABC transporters.

## Data Availability Statement

The original contributions presented in the study are included in the article/Supplementary Material, further inquiries can be directed to the corresponding author/s.

## Author Contributions

ZZ and JW conceived and designed the project. ZZ and JL performed the experiments. ZZ, JL, and JW wrote the manuscript. All authors contributed to the article and approved the submitted version.

## Conflict of Interest

The authors declare that the research was conducted in the absence of any commercial or financial relationships that could be construed as a potential conflict of interest.
