# Supplementary material for: DeepRTCP: Predicting ATP-Binding Cassette Transporters Based on 1-Dimensional Convolutional Network
Source: Front Cell Dev Biol. 2021 Feb 1;8:614080. doi: 10.3389/fcell.2020.614080 (PMC7882686; doi:10.3389/fcell.2020.614080)
Supplement: Supplementary file 1 [file Data_Sheet_1.PDF]

frontiers

Supplementary Material

1 SUPPLEMENTARY TABLES

1.1 Tables

Table S1. Performance comparison among SS_CP based RTCPs with different dimensions.

Dimensionality	Acc	Spec	F-score
236	92.77%	91.73%	0.9269
150	93.18%	92.16%	0.9310
120	93.20%	92.31%	0.9313
90	93.32%	92.31%	0.9323
80	93.25%	92.26%	0.9313

Table S2. Performance comparison among HP_CP based RTCPs with different dimensions.

Dimensionality	Acc	Spec	F-score
145	93.23%	93.23%	0.9421
120	93.01%	92.02%	0.9293
100	93.20%	92.31%	0.9313
90	93.06%	92.12%	0.9298
80	93.08%	92.16%	0.9300

Table S3. Performance comparison among HP_SS based RTCPs with different dimensions.

Dimensionality	Acc	Spec	F-score
145	93.07%	92.26%	0.9299
120	93.13%	92.31%	0.9307
80	93.44%	92.26%	0.9334
70	93.20%	92.40%	0.9113
50	93.34%	92.26%	0.9334

Table S4. Performance comparison among HP_ST based RTCPs with different dimensions.

Dimensionality	Acc	Spec	F-score
363	93.01%	91.69%	0.9290
300	93.30%	92.16%	0.9321
200	93.39%	92.11%	0.9329
100	93.54%	92.21%	0.9343
80	93.04%	91.54%	0.9284

Table S5. Performance comparison among ST_CP based RTCPs with different dimensions.

Dimensionality	Acc	Spec	F-score
363	92.69%	91.55%	0.9286
300	92.89%	91.50%	0.9321
200	93.39%	92.11%	0.9278
100	93.68%	92.31%	0.9357
80	93.13%	92.11%	0.9305

Table S6. Performance comparison among ST_SS based RTCPs with different dimensions.

Dimensionality	Acc	Spec	F-score
532	92.96%	91.88%	0.9288
300	92.47%	91.55%	0.9239
100	93.11%	92.26%	0.9303
80	93.94%	92.45%	0.9383
50	93.40%	92.12%	0.9279

Table S7. Performance comparison of 7-layers DCNN with different filter numbers.

Classifier	Train accuracy	Validation accuracy
8	94.80%	94.29%
16	96.19%	94.77%
24	98.23%	94.29%
32	99.26%	96.43%
40	99.37%	95.96%
48	99.31%	96.19%
56	99.57%	96.19%
64	99.28%	96.43%
72	99.34%	96.19%
80	99.52%	96.19%
